# Inflammasome-Independent Role for NLRP3 in Controlling Innate Antihelminth Immunity and Tissue Repair in the Lung

**DOI:** 10.4049/jimmunol.1900640

**Published:** 2019-10-04

**Authors:** Alistair L. Chenery, Rafid Alhallaf, Zainab Agha, Jesuthas Ajendra, James E. Parkinson, Martha M. Cooper, Brian H. K. Chan, Ramon M. Eichenberger, Lindsay A. Dent, Avril A. B. Robertson, Andreas Kupz, David Brough, Alex Loukas, Tara E. Sutherland, Judith E. Allen, Paul R. Giacomin

**Affiliations:** * Wellcome Centre for Cell-Matrix Research, Manchester M13 9PT, United Kingdom;; † Faculty of Biology, Medicine and Health, Manchester Academic Health Science Centre, University of Manchester, Manchester M13 9PL, United Kingdom;; ‡ Lydia Becker Institute for Immunology and Infection, Faculty of Biology, Medicine and Health, Manchester Academic Health Science Centre, University of Manchester, Manchester M13 9PL, United Kingdom;; § Centre for Molecular Therapeutics, Australian Institute of Tropical Health and Medicine, James Cook University, Smithfield, Queensland 4878, Australia;; ¶ Department of Molecular and Biomedical Science, School of Biological Sciences, University of Adelaide, Adelaide, South Australia 5000, Australia; and; ‖ School of Chemistry and Molecular Biosciences, University of Queensland, St Lucia, Queensland 4072, Australia

## Abstract

Alternatively activated macrophages are essential effector cells during type 2 immunity and tissue repair following helminth infections. We previously showed that Ym1, an alternative activation marker, can drive innate IL-1R–dependent neutrophil recruitment during infection with the lung-migrating nematode, *Nippostrongylus brasiliensis*, suggesting a potential role for the inflammasome in the IL-1–mediated innate response to infection. Although inflammasome proteins such as NLRP3 have important proinflammatory functions in macrophages, their role during type 2 responses and repair are less defined. We therefore infected *Nlrp3*^−/−^ mice with *N. brasiliensis*. Unexpectedly, compared with wild-type (WT) mice, infected *Nlrp3*^−/−^ mice had increased neutrophilia and eosinophilia, correlating with enhanced worm killing but at the expense of increased tissue damage and delayed lung repair. Transcriptional profiling showed that infected *Nlrp3*^−/−^ mice exhibited elevated type 2 gene expression compared with WT mice. Notably, inflammasome activation was not evident early postinfection with *N. brasiliensis*, and in contrast to *Nlrp3*^−/−^ mice, antihelminth responses were unaffected in caspase-1/11–deficient or WT mice treated with the NLRP3-specific inhibitor MCC950. Together these data suggest that NLRP3 has a role in constraining lung neutrophilia, helminth killing, and type 2 immune responses in an inflammasome-independent manner.

## Key Points

- *Nlrp3*^−/−^ mice have enhanced early antihelminth immunity in the lung.
- Type 2 immunity and repair responses are dysregulated in *Nlrp3^−/−^* mice.
- NLRP3 plays an inflammasome-independent role during *Nippostrongylus* infection.

## Introduction

The lungs are a vital barrier organ that must respond appropriately to both pathogens and innocuous Ags to maintain homeostasis. After tissue injury or an infectious insult, the lungs rapidly initiate immune resolution and repair mechanisms to maintain their essential physiological function. Alveolar macrophages (Mφs) help maintain homeostasis and can drive either proinflammatory or proresolution responses in the lung tissue microenvironment, depending on the stimulus. Classically activated Mφs upregulate antimicrobial factors such as NO, TNF-α, IL-6, and IL-1β in response to pathogen or damage-associated molecular patterns (1). Conversely, during helminth infections, Mφs can become alternatively activated in response to IL-4Rα signaling, upregulating arginase, resistin-like molecule (RELM)-α, chitinase-like protein Ym1, and specific matrix metalloproteinases (MMPs), all important type 2 effector molecules with wound healing functions (2–4). IL-4Rα–activated Mφs are known to mediate tissue repair in the skin (4) but also control airway inflammation and hemorrhage following the acute lung injury caused by primary infection with lung-migrating nematodes (5). Upon secondary infection, IL-4Rα–activated Mφs act in cooperation with antiparasitic neutrophil, eosinophil, type 2 innate lymphoid cell, and memory Th2 cell responses to mediate parasite control (6–9).

Mφ-derived Ym1 is a key feature of acute lung injury following infection with the nematode *Nippostrongylus brasiliensis*, with further increases in expression in response to the subsequent Th2 cell response (2). However, even in the absence of IL-4 under steady-state conditions, alveolar Mφs express substantial amounts of Ym1 (2). We have previously shown that as early as 2 d after *N. brasiliensis* infection, Ym1 plays a prominent role in the recruitment of neutrophils that swarm around larvae in the lungs and promote worm killing (10). IL-17A–producing γδ T cells are key to Ym1-mediated neutrophilia. Ectopic overexpression of Ym1 drives *Il1b* expression, whereas neutralizing Ym1 prevents expression of *Il1b* following *N. brasiliensis* infection (10). Because blockade of the IL-1R reduces the ability of Ym1 to induce IL-17–producing γδ T cells and associated neutrophilia (10), we and others (11) hypothesized that Ym1 may activate an inflammasome, leading to IL-1 release. In addition, Ym1 is well known to crystallize under conditions of chronic inflammation (12) and crystalline/particulate material can activate the NLR family member NACHT, LRR and PYD domains-containing protein 3 (NLRP3) inflammasome, suggesting that Ym1 may be a specific activator of NLRP3. Therefore, we predicted that NLRP3 inflammasome activation by alveolar Mφs would drive early neutrophilic responses important for lung-stage larval killing during *N. brasiliensis* infection.

Although the NLRP3 inflammasome has been widely studied in the context of classically activated Mφs, comparatively little is known about whether NLRP3 plays a role during alternative activation and type 2 inflammatory settings in which Ym1 is highly induced. Our previous studies show that NLRP3 constrains type 2 responses during infection with the gut-dwelling helminth parasite *Trichuris muris*, with a major effect on the development of adaptive Th2 cell immunity (13). In the current study, we addressed whether the NLRP3 inflammasome contributed to acute lung injury, innate immune cell activation, and lung repair during infection with *N. brasiliensis*. Counter to expectations, we discovered that *Nlrp3* deficiency promoted rather than constrained early lung neutrophilia. Additionally, lack of NLRP3 promoted host antihelminth effector mechanisms and other type 2 immune responses, but was detrimental with respect to infection-induced tissue damage.

## Materials and Methods

### Mice and ethics statements

*Nlrp3*^tm1Vmd^ (Research Resource Identifier [RRID]:MGI:5468973) (14) (University of Manchester), B6-*Nlrp3*^tm1Tsc^/Siec (RRID:IMSR_MUGEN:M153001) (James Cook University [JCU]), and *Casp1/11*^−/−^ (RRID:IMSR_JAX:016621) (JCU) mice were maintained on a C57BL/6J (RRID:IMSR_JAX:000664) background to generate littermate controls and bred in-house at the University of Manchester and JCU. All experiments were carried out in accordance with the U.K. Animals (Scientific Procedures) Act 1986 and under a Project License (70/8548) granted by the Home Office and approved by local Animal Ethics Review Group at the University of Manchester. Animal experimental protocols in Australia were approved by the JCU Animal Ethics Committee (A2213).

### *N. brasiliensis* infection

*N. brasiliensis* worms were propagated as previously described (15). Infective third-stage larvae were isolated and washed with sterile PBS and counted using a dissecting microscope. Mice were injected with 250 or 500 third-stage larvae s.c. Upon culling the mice by pentobarbitone overdose i.p., bronchoalveolar lavage (BAL) was performed with 10% FBS in PBS, and lung lobes were collected. Lobes were either stored in RNAlater (Thermo Fisher Scientific), fixed in 10% formalin for histology, or digested with Liberase TL (Roche). For lung-stage L4 counts, on days 1–2 postinfection, lungs were minced and incubated in PBS for 3 h at 37°C. Emergent larvae were counted using a dissecting microscope. For small intestinal worm burdens, worms were counted using a dissecting microscope following incubation at 37°C. Fecal parasite eggs were enumerated from one fecal pellet/animal collected 6 d postinfection using a Whitlock paracytometer.

### NLRP3 and caspase-1 inhibition

To inhibit NLRP3, 1 d prior to *N. brasiliensis* infection, mice were treated with either PBS vehicle or 10 mg/kg MCC950 (Sigma-Aldrich) by i.p. injection in a 100 μl volume, a dose that has been previously shown to inhibit inflammasome activity (16). To inhibit caspase-1, mice were treated with 25 mg/kg VX-765 (Generon) in a PBS vehicle containing 5% (v/v) DMSO/5% Kolliphor EL (v/v) (Sigma-Aldrich). Injections were repeated daily up until the experimental end point.

### IL-4 complex injection and in vitro IL-4 stimulation

For in vivo IL-4 stimulation, mice were injected i.p. with 1.5 μg rIL-4 (Miltenyi Biotec) complexed with 7.5 μg anti-IL-4 (Bio X Cell). After 24 h, lavage was performed to isolate peritoneal exudate cells containing predominantly Mφs, which were analyzed by flow cytometry. For in vitro stimulation, day 6 cultured bone marrow–derived Mφs (BMDMs, differentiated with L929-conditioned media) were treated with 20 ng/ml IL-4 and analyzed by flow cytometry the following day.

### Ex vivo caspase-1 activation assay

BAL cells were collected from mice at day 2 postinfection with *N. brasiliensis*. Cells were then stained for inflammasome activation using a caspase-1 FAM-YVAD-FMK FLICA probe kit (Bio-Rad). Cells were then processed for analysis by flow cytometry.

### Flow cytometry

Single-cell suspensions were washed in PBS, and Live/Dead staining (Thermo Fisher Scientific) was performed. Samples were Fc-blocked using anti-CD16/32 (2.4G2, RRID:AB_394656) (BD Biosciences) and mouse serum (Bio-Rad). Blocking and subsequent surface staining was performed using PBS containing 2 mM EDTA, 2% FBS, and 0.05% NaN_3_. Abs used for staining are listed in Table I. Following surface staining, cells were incubated with intracellular fixation buffer (Thermo Fisher Scientific) prior to permeabilization for intracellular staining. For secondary detection of Ym1 and RELM-α, Zenon goat (RRID:AB_2753200) and rabbit (RRID:AB_2572214) Ab labels (Thermo Fisher Scientific) were used. For Ym1, RELM-α, and pro–IL-1β intracellular staining, cells were directly stained without stimulation or protein transport inhibition. For cell quantification, some samples were spiked with 10 μm polystyrene beads (Sigma-Aldrich) prior to acquisition. Data were acquired on a BD LSRFortessa flow cytometer and analyzed using FlowJo v10 software.

### ELISA

BAL supernatants were analyzed for Ym1 using commercially available ELISA kits (R&D Systems). Lung homogenates were assayed for IL-4 using standard sandwich ELISA protocols (eBioscience). Analytes were detected using HRP-conjugated streptavidin and TMB substrate (BioLegend) and stopped with 1 M HCl. Final absorbance at 450 nm was measured using a VersaMax microplate reader (Molecular Devices).

### RNA extraction and quantitative real-time PCR

Tissue samples stored in RNAlater (Thermo Fisher Scientific) were processed for RNA extraction using a TissueLyser II and QIAzol reagent (Qiagen). Isolated RNA was quantified using a Qubit fluorimeter and RNA BR kit (Qiagen). cDNA was synthesized using Tetro reverse transcription kit (Bioline) and oligo dT 15-mers (Integrated DNA Technologies). Quantitative real-time PCR was performed using SYBR Green mix (Agilent Technologies) and a LightCycler 480 II (Roche). A list of primer sequences used is shown in Table II.

### Transcriptional profiling

Quality control was performed on RNA samples with an Agilent 2200 TapeStation system prior to downstream analyses. Samples were diluted and 100 ng of RNA was processed for running on a Nanostring nCounter FLEX system using the Myeloid Innate Immunity v2 panel. Please note that this panel does not distinguish *Chil3* from *Chil4*. Raw counts were normalized to internal spike-in controls and the expression of 13 stable housekeeping genes, as determined by geNorm algorithm within the nSolver Advanced Analysis tool. Subsequent analyses were performed in R (version 3.5.3). After normalization, transcripts with >15 counts were considered to be expressed and were log_2_ transformed. Linear modeling using the limma R package (17) was used to calculate differential gene expression. All expressed genes were used for principal component analysis (PCA). Unsupervised hierarchical clustering was performed on significantly differentially expressed genes between *N. brasiliensis*–infected wild-type (WT) and *Nlrp3*^−/−^ mice using the complete-linkage method and Euclidean distances.

### Histology and fractal analysis

Proximal small intestine was fixed in 4% neutral-buffered formaldehyde and embedded in paraffin, and 5-μM sections were stained with PAS/Alcian blue stains using the standard protocol of an institutional histology service provider (JCU). Goblet cells were quantified by counting PAS^+^ cells in 10 randomly selected villi units and averaged for each individual mouse. Whole left lung lobes were paraffin embedded, and 5-μm sections were prepared for hematoxylin/eosin staining and immunofluorescence staining. Slides were imaged using an Olympus or Leica slide scanner, and high-resolution image files were exported using Pannoramic Viewer software (3DHISTECH). The images were then processed in a KNIME software workflow to obtain 50 random regions of interests (ROIs) across the whole lung section. ROIs that contained lobe boundaries or extensive artifacts were excluded from the analysis. The ROIs were then converted to binary images, and lacunarity (Λ) was quantified using the FracLac plugin for ImageJ (default settings). The Λ values of all the ROIs were averaged to obtain estimates for the entire lobe.

### Statistical analyses

GraphPad Prism 7 software was used for all statistical analyses. Data were assessed to be normally distributed by the D’Agostino–Pearson omnibus normality test. Differences between experimental groups were assessed by ANOVA (for normally distributed data) followed by Tukey–Kramer post hoc multiple comparisons test or an unpaired two-tailed Student *t* test. In cases in which data were not normally distributed, a Kruskal–Wallis test was used. For gene expression data, values were log_2_ transformed to achieve normal distribution. Comparisons with a *p* value <0.05 were considered to be statistically significant.

## Results

### NLRP3 deficiency enhances early innate immune cell recruitment to the lung

**FIGURE 1.**
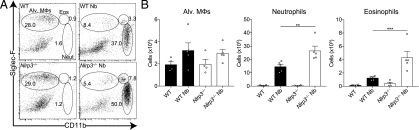
*Nlrp3* deficiency increases lung innate cell recruitment during *N. brasiliensis* infection. WT and *Nlrp3*^−/−^ mice were infected with *N. brasiliensis* (Nb) and day 2 postinfected lung alveolar (alv.) Mφ (CD11b^lo^Siglec-F^+^CD11c^+^), neutrophil (CD11b^+^Siglec-F^−^Ly6G^+^), and eosinophil (CD11b^+^Siglec-F^+^) (**A**) frequencies of live cells and (**B**) absolute numbers were measured by flow cytometry. Data are representative (mean ± SEM) of four individual experiments with three to five mice per group (per experiment). **p < 0.01, ***p < 0.001, one-way ANOVA and Tukey–Kramer post hoc test).

To test our hypothesis that NLRP3 is required for the early recruitment of neutrophils into the lung during infection with lung-migrating helminths, WT and *Nlrp3*^−/−^ mice were infected with *N. brasiliensis*. Lung tissue cells were isolated and analyzed on day 2 postinfection, at a peak of injury and neutrophilia (Table I). As expected, *N. brasiliensis* infection of WT mice led to increased frequencies (Fig. 1A) and total numbers (Fig. 1B) of eosinophils and neutrophils in the lung, compared with naive mice. Unexpectedly, infected *Nlrp3*^−/−^ mice had increased numbers of recruited neutrophils and eosinophils compared with infected WT mice. Total numbers of alveolar Mφs were not significantly different between all groups (Fig. 1A, 1B). Cellular content of BAL fluid was not analyzed because all infected samples on day 2 were bloody because of lung damage. Together, these data suggest that in contrast to the canonical role for NLRP3 in promoting granulocytic inflammation via inflammasome activation, NLRP3 may actually have a role in restraining granulocyte recruitment in type 2 settings.

**Table I.** List of flow cytometry Abs used

Ag	Clone	Manufacturer	RRID
CD11b	M1/70	BioLegend	AB_11218791
CD11c	N418	BioLegend	AB_493569
Ly6C	HK1.4	BioLegend	AB_1134213
Tim4	RMT4-54	BioLegend	AB_2565718
CD4	GK1.5	BioLegend	AB_10900241
CD8	53-6.7	BioLegend	AB_2075239
CD19	6D5	BioLegend	AB_11203527
TCRβ	H57-597	BioLegend	AB_893625
TCRγδ	GL3	BioLegend	AB_313832
ST2	DIH9	BioLegend	AB_2565634
IL-5	TRFK5	BioLegend	AB_315328
IL-17A	TC11-18H10.1	BioLegend	AB_536018
Ly6G	1A8	BD Biosciences	AB_394208
Siglec-F	E50-2440	BD Biosciences	AB_2722581
CD3ε	17A2	Thermo Fisher Scientific	AB_467055
F4/80	BM8	Thermo Fisher Scientific	AB_10372666
B220	RA3-6B2	Thermo Fisher Scientific	AB_1957381
pro–IL-1β	NJTEN3	Thermo Fisher Scientific	AB_2573995
IL-13	eBio13A	Thermo Fisher Scientific	AB_2535336
Ym1	Polyclonal	R&D Systems	AB_2260451
RELM-α	Polyclonal	PeproTech	AB_1621941

### Nlrp3^−/−^ mice have increased innate antihelminth immunity in the lung

**FIGURE 2.**
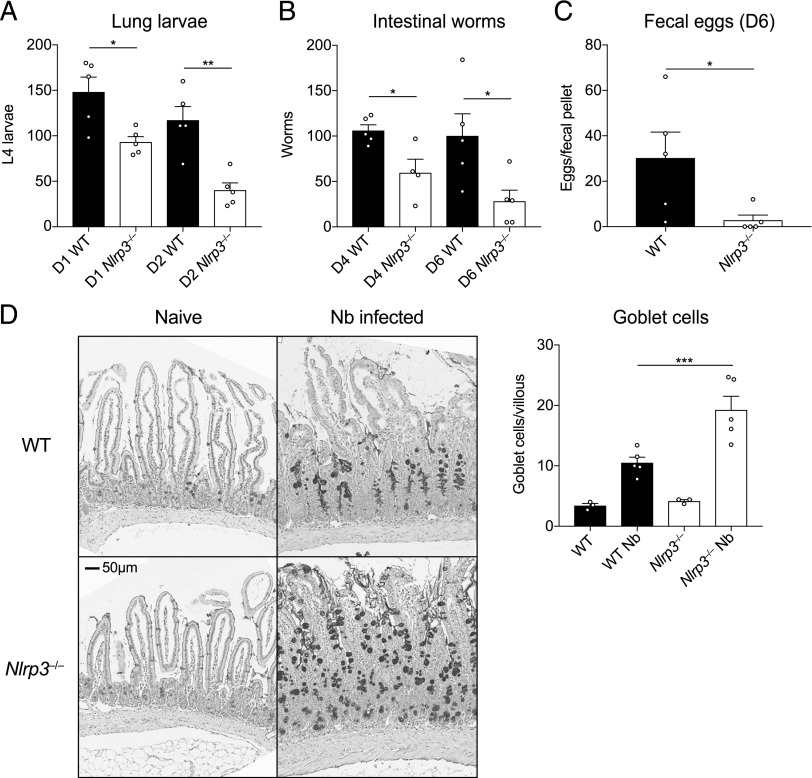
Enhanced antihelminth responses in the absence of NLRP3. WT and *Nlrp3*^−/−^ mice were infected with 500 *N. brasiliensis* (Nb) L3s, and (**A**) lung stage L4 larvae were quantified on days 1 and 2 postinfection. (**B**) Adult worms in the small intestine (days 4 and 6 postinfection) and (**C**) fecal parasite eggs shed in the stool (day 6) were quantified (per fecal pellet). (**D**) Representative images of proximal small intestine from naive and day 6 infected mice, stained with Alcian blue/periodic acid-Schiff. Goblet cells were enumerated from 10 randomly selected villi per mouse. Data from (A)–(D) are representative (mean ± SEM) of three individual experiments with three to five mice per group (per experiment). *p < 0.05, **p < 0.01, ***p < 0.001, one-way ANOVA and Tukey–Kramer post hoc test or unpaired two-tailed Student *t* test.

We next addressed whether the increased granulocyte infiltration in infected *Nlrp3*^−/−^ mice correlated with increased antihelminth immunity. Rapid neutrophil recruitment to the lung in particular is known to be important for immunity to *N. brasiliensis* (5, 10). Thus, we assessed numbers of larvae in WT and *Nlrp3*^−/−^ mice during primary *N. brasiliensis* infection. Consistent with the enhanced early granulocyte response (Fig. 1B), *Nlrp3*^−/−^ mice displayed reduced numbers of lung-stage L4 larvae at both day 1 and day 2 postinfection compared with WT mice (Fig. 2A). Consequently, *Nlrp3*^−/−^ mice also displayed less parasite migration to the intestine at day 4 and day 6 postinfection (Fig. 2B) and a decrease in shed fecal eggs (Fig. 2C). Although we saw no differences in goblet cell numbers under basal conditions, *Nlrp3*^−/−^ mice also displayed increased presence of goblet cells in the small intestine postinfection (Fig. 2D), consistent with an increased effector type 2 response in which goblet cell hyperplasia correlates with intestinal worm expulsion (18). Together, these data suggest that lack of NLRP3 leads to increased protective innate immune responses in the lung following *N. brasiliensis* infection, potentially by promoting neutrophil and eosinophil accumulation.

### NLRP3 regulates lung repair after helminth infection

**FIGURE 3.**
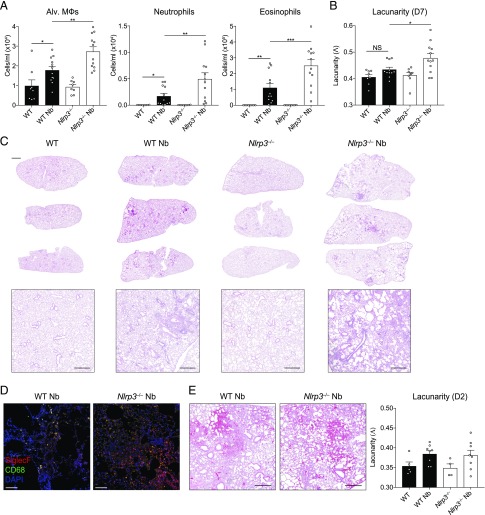
NLRP3 regulates lung tissue repair following *N. brasiliensis* infection. WT and *Nlrp3*^−/−^ mice were infected with *N. brasiliensis* (Nb), and (**A**) day 7, postinfected BAL alveolar (alv.) Mφ (CD11b^−^Siglec-F^+^CD11c^+^), neutrophil (CD11b^+^Siglec-F^−^Ly6G^+^), and eosinophil (CD11b^+^Siglec-F^+^) absolute numbers were measured by flow cytometry. H&E staining was performed on lung sections and imaged followed by (**B**) quantification of lacunarity (Λ) to assess lung damage on day 7. (**C**) Representative image insets are shown on top with low magnification scans of entire left lung lobes from individual mice shown below (top panel scale bar, 1000 μm; bottom panel scale bar, 500 μm). (**D**) Immunofluorescence staining of lung inflammatory foci with SiglecF (red), CD68 (green), and DAPI (blue) (scale bar, 100 μm). (**E**) Representative day 2 postinfection lung sections and lacunarity (scale bar, 500 μm). Data were pooled (A, B, and E; mean ± SEM) from three individual experiments with three to five mice per group (per experiment). *p < 0.05, **p < 0.01, ***p < 0.001, one-way ANOVA and Tukey–Kramer post hoc test.

The data thus far demonstrated that *Nlrp3*^−/−^ mice displayed elevated neutrophilia and eosinophilia in the lung and protective immunity in the early stages of primary infection, accompanied by an enhanced type 2 effector response in the intestine. Both types of granulocytes may contribute to lung tissue damage during *N. brasiliensis* infection and allergic responses (5, 10), but type 2 responses are typically tissue protective (19). It was therefore important to assess whether elevated granulocytic responses in *Nlrp3*^−/−^ mice impacted the resolution of inflammation and repair of lung tissue damage during the later stage of the infection model (20, 21). To assess airway cell infiltration, BAL was performed on mice at day 7 after *N. brasiliensis* infection, at a timepoint when hemorrhaging has normally ceased and larvae have completely exited the lung tissue. As expected, eosinophils were the predominant cell type elevated in the airways of infected WT mice compared with naive controls along with increased alveolar Mφs and neutrophils (Fig. 3A). Critically, all three cell types were significantly elevated in infected *Nlrp3*^−/−^ mice compared with infected WT mice, suggesting a persistence of cellular inflammation in the airways. This was consistent with evidence of increased lung tissue damage in *Nlrp3*^−/−^ mice compared with WT mice postinfection (Fig. 3B, 3C). Specifically, whereas lungs from infected WT mice displayed modest airway cell infiltration and features of successful tissue repair, *Nlrp3*^−/−^ lungs had larger and more numerous immune cell foci and larger alveolar spaces indicating impaired repair (Fig. 3C). The inflammatory foci in the *Nlrp3*^−/−^ mouse lungs were comprised of a mixture of myeloid cells with a large presence of CD68^−^SiglecF^+^ eosinophils and CD68^+^SiglecF^+^ Mφs (Fig. 3D). To quantify lung damage, we performed fractal/lacunarity analysis of entire lung lobes, which has previously been shown to robustly measure lung damage, correlating well with traditional stereological measurements such as mean linear intercept (22). Lacunarity (Λ), a measure of heterogeneity/gaps in a structure that correlates with airway damage, was increased for infected *Nlrp3*^−/−^ lungs compared with infected WT lungs, which showed no significant differences over WT naive controls (Fig. 3B). We did not detect differences in tissue damage between WT and *Nlrp3*^−/−^ lungs as early as day 2 postinfection (Fig. 3E). Together, these data reveal that NLRP3 is required for the resolution of inflammation and the timely initiation of repair processes in the lung following injury by helminth infection.

### Nlrp3^−/−^ mice have enhanced type 2 cytokine responses and Ym1 expression

**FIGURE 4.**
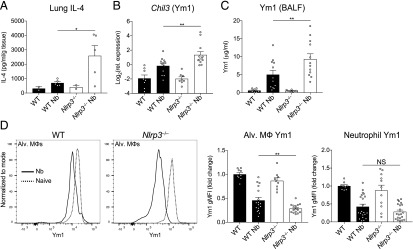
NLRP3 regulates Ym1 following infection with *N. brasiliensis*. Postinfection of WT and *Nlrp3*^−/−^ mice with *N. brasiliensis* (Nb), (**A**) IL-4 protein levels were measured in lung tissues by ELISA on day 2 postinfection (normalized to tissue weight). (**B**) *Chil3* (Ym1) gene expression was measured by qRT-PCR on day 7 postinfection (log_2_ expression relative to *Rpl13a*). (**C**) BALF Ym1 was measured by ELISA and (**D**) intracellular expression was measured by gMFI (fold change over WT control) by alveolar (alv.) Mφs, and neutrophils were quantified by flow cytometry on day 7 postinfection. Data are representative (A; mean ± SEM) or pooled (B–D; mean ± SEM) from three individual experiments with three to five mice per group (per experiment). *p < 0.05, **p < 0.01, one-way ANOVA and Tukey–Kramer post hoc test.

The failure of *Nlrp3*^−/−^ mice to repair as well as WT mice could reflect a deficient type 2 immune response. However, *Nlrp3*^−/−^ mice exhibited enhanced features of type 2 immunity, including lung eosinophilia and increased intestinal goblets cells. Lung tissue repair following *N. brasiliensis* infection is dependent on type 2 responses, including the expansion of IL-4Rα–activated Mφs (5). Critically, whereas innate sources of Ym1 induce neutrophilia (10), during the later reparative stage, IL-4Rα–induced Ym1 has a direct role in repair (2). We therefore examined whether type 2 responses known to be involved in repair are dysregulated in *Nlrp3*^−/−^ mice during *N. brasiliensis* infection. Despite a failure to repair, compared with infected WT mice, infected *Nlrp3*^−/−^ mice exhibited enhanced IL-4 protein levels in the lungs as early as day 2 postinfection (Fig. 4A). By day 7 postinfection, using quantitative RT-PCR (qRT-PCR) (Table II), we found that *Chil3* (Ym1) expression was significantly increased in the lungs of *Nlrp3*^−/−^ mice compared with WT mice (Fig. 4B). Similarly, there were significant increases in secreted Ym1 protein levels in the BAL fluid of infected *Nlrp3*^−/−^ mice (Fig. 4C). To define the cellular source of Ym1, we performed intracellular cytokine staining and flow cytometry focusing on alveolar Mφs and neutrophils that are known sources of Ym1 during *N. brasiliensis* infection (2). We found that infection caused a drop in the geometric mean fluorescence intensity (gMFI) of Ym1 staining in both alveolar Mφs and neutrophils (Fig. 4D), consistent with release of Ym1 into the airways, as observed in the BAL fluid (Fig. 4C). However, *Nlrp3*^−/−^ alveolar Mφs had a greater drop in gMFI for Ym1 following infection compared with WT alveolar Mφs. Conversely, Ym1 release by neutrophils was equivalent in infected WT and *Nlrp3*^−/−^ mice. Therefore, NLRP3 deficiency influences expression of IL-4 and Ym1 in the lung during infection with *N. brasiliensis* and may control release of Ym1 by alveolar Mφs in the airways.

**Table II.** List of primer sequences used

Primer	Sequence (5′-3′)
*Arg1* forward	5′-CTCCAAGCCAAAGTCCTTAGAG-3′
*Arg1* reverse	5′-AGGAGCTGTCATTAGGGACATC-3′
*Ccl24* forward	5′-CAGCCTTCTAAAGGGGCCAA-3′
*Ccl24* reverse	5′-GGTCTGTCAAACCCCAAAGC-3′
*Chil3* forward	5′-ACCTGCCCCGTTCAGTGCCAT-3′
*Chil3* reverse	5′-CCTTGGAATGTCTTTCTCCACAG-3′
*Cxcl3* forward	5′-GGTTGATTTTGAGACCATCCAG-3′
*Cxcl3* reverse	5′-CTTCTTGACCATCCTTGAGAGT-3′
*Ear6* forward	5′-GTAACCTCACAACTCCGAGAAGA-3′
*Ear6* reverse	5′-TGCTGGCACTGGAGCTAAAAT-3′
*Il4* forward	5′-CCTGCTCTTCTTTCTCGAATGT-3′
*Il4* reverse	5′-CACATCCATCTCCGTGCAT-3′
*Mmp13* forward	5′-CTTCTTCTTGTTGAGCTGGACTC-3′
*Mmp13* reverse	5′-CTGTGGAGGTCACTGTAGACT-3′
*Retnla* forward	5′-TATGAACAGATGGGCCTCCT-3′
*Retnla* reverse	5′-GGCAGTTGCAAGTATCTCCAC-3′
*Rpl13a* forward	5′-CATGAGGTCGGGTGGAAGTA-3′
*Rpl13a* reverse	5′-GCCTGTTTCCGTAACCTCAA-3′

### Type 2 gene expression is dysregulated in Nlrp3^−/−^ mice

**FIGURE 5.**
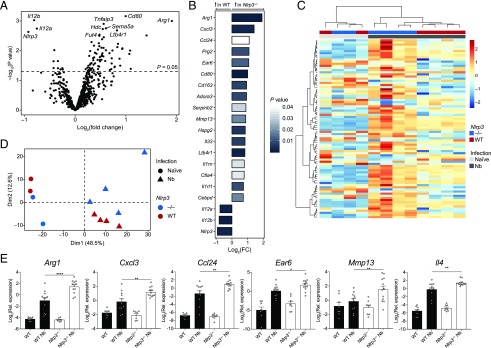
Dysregulated type 2 gene expression in *Nlrp3*^−/−^ mice following *N. brasiliensis* infection. Whole lung RNA from WT and *Nlrp3*^−/−^ mice on day 7 postinfection with *N. brasiliensis* (Nb) was analyzed by NanoString. (**A**) Volcano plot showing differentially expressed genes between *N. brasiliensis*–infected WT and *Nlrp3*^−/−^ mice. (**B**) Top 20 differentially regulated genes between *N. brasiliensis*–infected WT and *Nlrp3*^−/−^ mice (bar color indicates statistical significance). (**C**) Unsupervised, hierarchically clustered heatmap of genes differentially expressed 7 d after *N. brasiliensis* infection in WT and *Nlrp3*^−/−^ mice. (**D**) PCA of naive and *N. brasiliensis*–infected WT and *Nlrp3*^−/−^ mice. (**E**) Candidate differentially expressed genes validated by qRT-PCR (log_2_ expression relative to *Rpl13a*). Data were from a single Nanostring run (A–D; *n* = 2–4 mice per group) or pooled (E; mean ± SEM) from three individual experiments with three to five mice per group (per experiment). *p < 0.05, **p < 0.01, ****p < 0.0001, one-way ANOVA and Tukey–Kramer post hoc test. Dim, dimension.

To more broadly characterize the immune processes potentially dysregulated in *Nlrp3*^−/−^ mice, we performed differential gene expression analysis of whole lung RNA on day 7 postinfection with *N. brasiliensis* using the NanoString nCounter Myeloid Innate Immunity panel. We observed differential expression of well-characterized genes associated with the later stages of *N. brasiliensis* infection such as *Rnase2a*, *Retnla*, and *Chil3/4* that were, as expected, highly upregulated in infected WT mice relative to uninfected mice (Supplemental Fig. 1A). Under basal conditions, few genes were found to be downregulated in *Nlrp3*^−/−^ mice compared with naive WT mice (Supplemental Fig. 1A). However, in *N. brasiliensis*–infected mice, 84 genes were differentially expressed between *Nlrp3*^−/−^ mice compared with WT (*p* < 0.05) (Fig. 5A, 5B). Although no genes remained statistically significant after Benjamini–Hochburg multiple test correction (23), dimensionality reduction by PCA and hierarchical clustering (Fig. 5C, 5D) showed clear separation of gene expression patterns between *N. brasiliensis*–infected WT and *Nlrp3*^−/−^ mice. Dimension 1 represents the first principal component and accounted for the largest proportion of the total variation within the samples and clearly separated the samples by infection status. Further separation of samples by genotype is shown along Dimension 2, which represents the second principal component (Fig. 5D). Increased sample numbers were used to validate specific genes by qRT-PCR. Infected *Nlrp3*^−/−^ mice had significantly higher expression of the type 2 markers *Arg1* and *Ccl24* as well as an increase in the neutrophil-associated *Cxcl3* compared with infected WT mice (Fig. 5E). Despite not reaching significance in the NanoString analysis, qRT-PCR analysis showed that *Il4* expression was found to be significantly increased in infected *Nlrp3*^−/−^ mice compared with infected WT mice, confirming the protein levels observed in Fig. 4A. Additional genes whose expression was increased in infected *Nlrp3*^−/−^ mice included the matrix metalloprotease-encoding *Mmp13* and the alveolar Mφ-expressed gene *Ear6* (24). Thus, mice lacking NLRP3 have dysregulated type 2 gene expression in the lung following tissue injury caused by helminth infection and may have abnormal neutrophil chemotaxis and alveolar Mφ function.

### Tissue resident Mφ alternative activation is enhanced in Nlrp3^−/−^ mice

**FIGURE 6.**
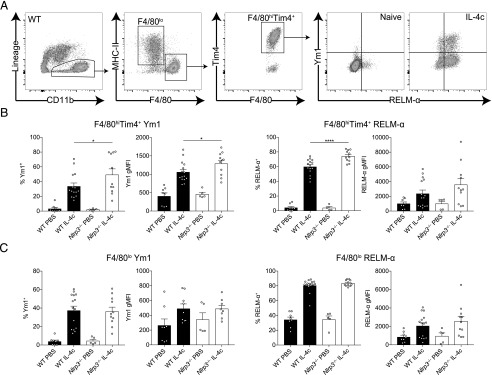
NLRP3 regulates resident Mφ alternative activation by IL-4. WT and *Nlrp3*^−/−^ mice were injected with either PBS or IL-4c, and (**A**) peritoneal CD11b^+^ cells containing F4/80^lo^ and F4/80^hi^Tim4^+^ Mφs where analyzed by flow cytometry after 24 h. Frequency and gMFI of intracellular Ym1 and RELM-α were determined in (**B**) resident F4/80^hi^Tim4^+^ Mφs and (**C**) monocyte-derived F4/80^lo^ Mφs. Data are representative (B, left panel; mean ± SEM) or pooled (B, right panel, and C; mean ± SEM) from three individual experiments with three to five mice per group (per experiment). *p < 0.05, ****p < 0.0001, one-way ANOVA and Tukey–Kramer post hoc test.

Our finding that expression of *Chil3* (Ym1) was higher in *N. brasiliensis*–infected *Nlrp3*^−/−^ mice (Fig. 4) is likely due to the enhanced quantities of IL-4 observed, as several IL-4–responsive genes were observed to be upregulated in the absence of NLRP3 (Fig. 5). However, to test the possibility that NLRP3 has the capacity to enhance Mφ responsiveness to IL-4Rα signaling, we used a reductionist model, in which IL-4 complex (IL-4c) delivery into the peritoneal cavity robustly induces expression of Ym1 and RELM-α in peritoneal Mφs (25). WT and *Nlrp3*^−/−^ mice were injected with IL-4c i.p., following which we analyzed the CD11b^+^ myeloid compartment containing F4/80^lo^ monocyte-derived Mφs and F4/80^hi^Tim4^+^ tissue resident Mφs after 24 h (Fig. 6A). There would be no expectation that IL-4c delivery would induce the NLRP3 inflammasome, and indeed, we were unable to detect any evidence for inflammasome activation in the peritoneal Mφs (data not shown). As expected, both Mφ populations showed increased intracellular expression of Ym1 and RELM-α in WT mice after IL-4c injection. IL-4c–stimulated *Nlrp3*^−/−^ resident (F4/80^hi^Tim4^+^) Mφs had greater frequencies of both Ym1^+^ and RELM-α^+^ cells as well as an increased Ym1 gMFI compared with IL-4c–stimulated WT resident Mφs (Fig. 6B). In contrast, we did not see enhanced expression of Ym1 or RELM-α above WT levels in *Nlrp3*^−/−^ monocyte-derived (F4/80^lo^) Mφs after IL-4c stimulation (Fig. 6C). Similarly, in vitro IL-4–stimulated BMDMs from *Nlrp3*^−/−^ mice did not show increased alternative activation markers compared with WT BMDMs (Supplemental Fig. 1B). Thus, it appears that in vivo NLRP3 presence versus absence influences the Mφ response to IL-4, independent of the inflammasome, but these effects may be restricted to specific Mφs subpopulations, such as tissue resident rather than monocyte-derived Mφs.

### Potential inflammasome-independent role of NLRP3 during lung antihelminth responses

**FIGURE 7.**
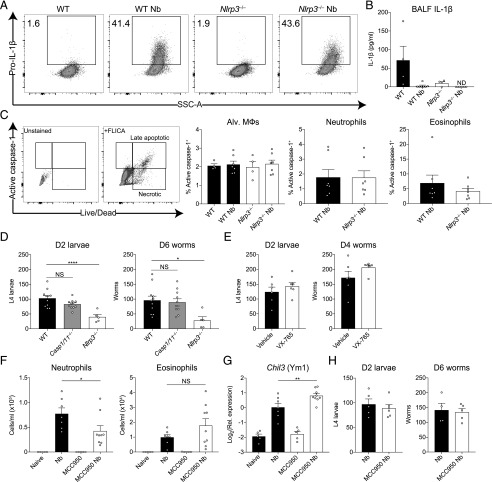
Inflammasome-independent role for NLRP3 during innate antihelminth responses in the lung. WT and *Nlrp3*^−/−^ mice were infected with *N. brasiliensis* (Nb), and on day 2 postinfection (**A**), intracellular pro–IL-1β levels were measured in alveolar (Alv.) Mφs by flow cytometry, (**B**) released IL-1β in the BALF was quantified by ELISA, and (**C**) frequency of active caspase-1 was measured ex vivo in BAL alveolar Mφs, neutrophils, and eosinophils by FAM-FLICA fluorescence. (**D**) Day 2 postinfection L4 lung-stage larvae and day 6 adult intestinal worms were counted in WT, *Casp1/11*^−/−^, and *Nlrp3*^−/−^ mice. (**E**) WT mice were treated with the caspase-1 inhibitor VX-765, and lung (day 2) and intestinal (day 4) worm burdens were measured. WT mice were treated with the NLRP3 inhibitor MCC950 during *N. brasiliensis* infection, and (**F**) BAL neutrophils and eosinophils were quantified and (**G**) *Chil3* (Ym1) expression in the lung was measured by qRT-PCR. (**H**) Lung larval burdens (day 2) and intestinal (day 6) worm burdens were counted following MCC950 treatment and *N. brasiliensis* infection. Data are representative (A–C and H; mean ± SEM) or pooled (D–G; mean ± SEM) from two or three individual experiments with three to six mice per group (per experiment). *p < 0.05, **p < 0.01, ****p < 0.0001, one-way ANOVA and Tukey–Kramer post hoc test.

Thus far we have demonstrated that although type 2 immune responses were enhanced in the absence of NLRP3, lung inflammation persisted after resolution of helminth infection. Our observation that the early neutrophilic response was enhanced in NLRP3-deficient mice was contrary to the expectation that infection with *N. brasiliensis* would result in inflammasome activation. Although NLRP3 is most commonly associated with forming an inflammasome, there are some reports of inflammasome-independent roles for NLRP3 (26, 27). Therefore, we investigated whether NLRP3 played inflammasome-dependent or -independent functions during *N. brasiliensis* infection. First, at 48 h postinfection during the peak of the lung injury, we examined expression of infection-induced production of pro–IL-1β within alveolar Mφs, a precursor for inflammasome activation. *N. brasiliensis* infection caused increased levels of pro–IL-1β within alveolar Mφs; however, this infection-induced response was unaffected by NLRP3 deficiency (Fig. 7A). Furthermore, levels of mature IL-1β released into the airways (an indicator of inflammasome activation) were nearly undetectable in the BAL fluid in both WT and *Nlrp3*^−/−^ mice (Fig. 7B), suggesting that inflammasome activation in the lung is not a major feature of *N. brasiliensis* infection. Similar results were observed at 24 h postinfection (data not shown). To further delineate whether inflammasome activation occurred following *N. brasiliensis* infection, we assessed the protease caspase-1. The inflammasome recruits and activates caspase-1 (28), which subsequently cleaves pro–IL-1β into its mature form (29). Activated caspase-1 was quantified ex vivo using a FAM-YVAD-FMK fluorochrome inhibitor of caspases (FLICA) probe on BAL cells on day 2 postinfection. Critically, there was no evidence of infection-induced increases in caspase-1 activity in alveolar Mφs (Fig. 7C). Similarly, we observed no differences in caspase-1 activity within neutrophils and eosinophils between infected WT and *Nlrp3*^−/−^ mice.

To more definitively establish whether inflammasome activation contributed to the observed phenotype of *Nlrp3*^−/−^ mice, we compared the response to *N. brasiliensis* infection in *Nlrp3*^−/−^ mice with that of *Casp1/11*^−/−^ mice, which cannot mount either canonical or noncanonical NLRP3 inflammasome responses (30). Lung-stage larvae and adult worm burdens were equivalent in *Casp1/11*^−/−^ and WT mice (Fig. 7D). In contrast, *Nlrp3*^−/−^ mice displayed increased antiparasitic immunity at the lung stage, characterized by significantly reduced lung larval numbers and adult worm burdens. Similarly, WT mice treated with the caspase-1 inhibitor VX-765 had no change in worm burdens (Fig. 7E). In a final effort to establish a role for *N. brasiliensis*–induced inflammasome activation, we treated WT mice with MCC950, an NLRP3-specific inflammasome small molecule inhibitor (31), throughout the course of infection. We failed to see changes in myeloid cell numbers in the BAL throughout the infection following MCC950 treatment (data not shown). By day 5 postinfection, we could see that mice treated with MCC950 had a reduction in BAL neutrophil numbers but had no change in eosinophil numbers (Fig. 7F). However, we did observe increased expression of *Chil3* in the lungs of infected mice treated with MCC950 (Fig. 7G). Although consistent with our findings in infected *Nlrp3*^−/−^ mice, other type 2 markers including IL-4 itself were unchanged in MCC950-treated or *Casp1/11*^−/−^ mice (data not shown). Furthermore, in contrast to NLRP3 deficiency, there was no difference in lung-stage larvae numbers or intestinal worm burden between untreated and MCC950-treated mice (Fig. 7H). Together, these data strongly suggest that NLRP3 plays an inflammasome-independent role in regulating neutrophil and antihelminth immunity in the lung.

## Discussion

Although the role of NLRP3 in classical inflammatory settings has been well characterized, the function of NLRP3 during type 2 inflammation has been more contentious. An early controversy focused on whether the type 2–promoting adjuvant activity of alum is dependent on its ability to activate the NLRP3 inflammasome (32, 33). Subsequently, a variety of studies have found that NLRP3 either promotes (34–36) or restricts (30, 37) allergic responses and Th2 cells. Most recently, Persson et al. (38) demonstrated a profound ability of Charcot–Leyden crystals to promote type 2 immunity but the ability of the crystals to activate the NLRP3 inflammasome was not required for the adjuvant effect. Thus, the role of NLRP3 in type 2 immunity remains largely unresolved.

To help resolve this question, we have focused on helminth infections, in which type 2 immunity is central for host protection. We previously found that the NLRP3 inflammasome played a major role in suppressing the adaptive type 2 immune response to intestinal helminth infection (in the cecum) with *T. muris* (13). In that study, MCC950 treatment and caspase-1/11 deficiency was able to phenocopy *Nlrp3*^−/−^ mice during infection. In contrast, the current study showed that during the innate immune phase of *N. brasiliensis* infection, NLRP3 had an unexpected, and apparently inflammasome-independent, role in constraining the initial neutrophilia as well as the eosinophilia that results from the type 2 responses to the parasite and subsequent expulsion in the small intestine. These data suggest that the NLRP3 inflammasome has differential roles during innate and adaptive immunity and may be tissue-specific, acting differently in two very distinct helminth infection models.

We previously published that Ym1 was responsible for a more aggressive early neutrophilic response in the lung, which promotes *N. brasiliensis* larval killing but at the cost of increased host tissue damage (10). Ym1 can form crystals in Mφs (12), which may be a trigger for NLRP3 inflammasome assembly (38). Classically, neutrophilic influx, which can be induced by the presence of necrotic cells and damage-associated molecular patterns such as extracellular ATP during acute tissue injury, has been shown to be critically driven by NLRP3 (39). Additionally, NLRP3 inflammasome-dependent release of IL-1β is critical for neutrophil antibacterial responses to *Streptococcus pneumoniae* infection in the lungs (40). We therefore hypothesized that Ym1 acted via NLRP3, and NLRP3 deficiency would reverse Ym1-mediated neutrophil recruitment. However, our data counterintuitively showed that during acute lung injury with *N. brasiliensis* infection, neutrophilia was enhanced in the absence of NLRP3. In addition, we saw no evidence for inflammasome activation during innate responses, and neither MCC950-mediated inflammasome inhibition nor caspase-1/11 deficiency replicated NLRP3 deficiency during *N. brasiliensis* infection. It is therefore possible that NLRP3 has distinct roles regulating neutrophil recruitment that can either be dependent or independent of inflammasome activation. Our data are consistent with another report in which NLRP3, but not caspase-1/11 or ASC, limited early neutrophil influx during lung bacterial infection with *Francisella tularensis* (27). CXCL3, a neutrophil chemotactic factor that can mobilize neutrophils from the bone marrow (41), was increased in the lungs of *N. brasiliensis*–infected NLRP3-deficient mice, suggesting a potential mechanism for NLRP3-dependent regulation of neutrophil recruitment. However, the increased *Cxcl3* expression we observed may just reflect increased neutrophil numbers as recruited neutrophils can express *Cxcl3* (24).

In line with previous studies (5, 10), the enhanced neutrophilia we observed in the absence of NLRP3 resulted in increased lung damage and impaired repair mechanisms. The initiation of repair responses in the lung following injury from *N. brasiliensis* migration is dependent on IL-4Rα signaling (5). Our transcriptional analysis revealed enhanced alternative Mφ activation and other type 2 markers such as *Il4* and *Arg1* in infected *Nlrp3*^−/−^ mice relative to WT controls. This correlated with enhanced *Ccl24* (Eotaxin-2) expression and increased eosinophilia in infected *Nlrp3*^−/−^ mice. Furthermore, *Mmp13* expression was also elevated in infected *Nlrp3*^−/−^ mice. As MMP13 has been shown to be regulated by IL-4Rα during *N. brasiliensis* infection (5), the data further highlight possible type 2–dependent defects in tissue repair processes in the absence of NLRP3. Thus, in our model NLRP3 deficiency enhances the host-protective type 2 response, and thus, one might expect enhanced repair. However, paradoxically there was persistent damage in the gene deficient mice, suggesting NLRP3 is required for the timely resolution of the early inflammatory response. It is possible that type 2 responses are exaggerated in the absence of NLRP3 because of a compensatory need to repair the damaged tissues. Our data may also fit the hypothesis that early neutrophil responses are required to establish a type 2 response (42) and because we saw enhanced neutrophilia in NLRP3-deficient mice, the magnitude of the resulting type 2 response could be exacerbated.

These findings highlight a complex relationship between NLRP3 and IL-4. It has been previously reported that IL-4 signaling negatively regulates NLRP3 by suppressing inflammasome assembly and caspase-1 activity as well as the subcellular localization of NLRP3 (43). Additionally, in CD4^+^ T cells it has been shown that NLRP3 can act as a transcription factor that supports differentiation to the Th2 cell lineage in an inflammasome-independent manner (26). Whether NLRP3 is acting as a transcription factor in Mφs similar to its action in Th2 cells remains to be determined. Our data in peritoneal Mφs provide evidence that NLRP3 can negatively regulate Mφ IL-4 responsiveness, independent of the inflammasome. However, we have yet to establish if there is an inflammasome-independent role for NLRP3 in alternative activation of the distinct lung Mφ populations, which vary in their response to IL-4 (44). Additionally, in our model, NLRP3 could be playing key roles in other lung myeloid cells such as neutrophils, monocytes, and dendritic cells, which cannot be excluded from contributing to the NLRP3 deficient phenotype. It will be important to clarify how inflammasome-dependent and -independent roles for NLRP3 relate to innate versus adaptive immune responses within type 2 disease contexts.

## Supplementary Material

Data Supplement
