# Supplementary material for: Inflammasome-Independent Role for NLRP3 in Controlling Innate Antihelminth Immunity and Tissue Repair in the Lung
Source: J Immunol. 2019 Oct 4;203(10):2724–34. doi: 10.4049/jimmunol.1900640 (PMC6826118; doi:10.4049/jimmunol.1900640)
Supplement: Data Supplement [file JI_1900640.zip › JI_1900640_Supplemental_Figure_1.pdf]

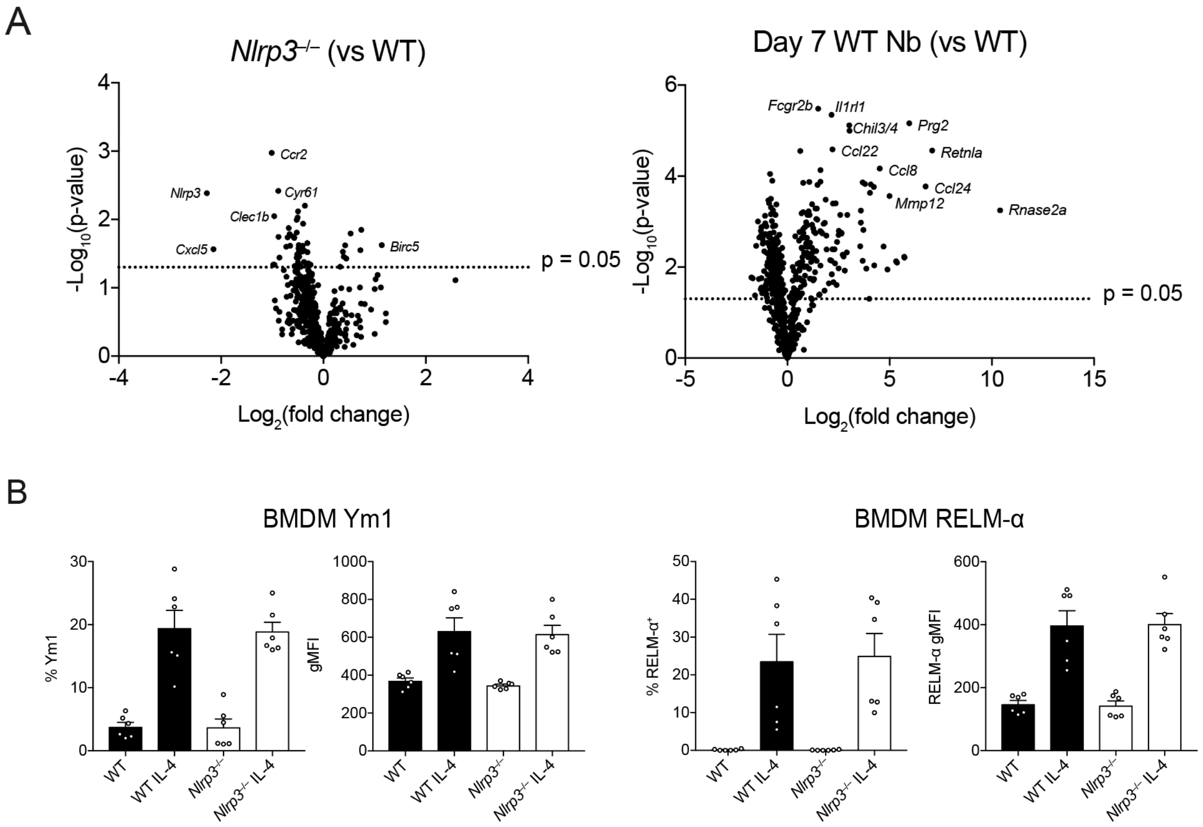

Supplemental Figure 1 (**A**) Differential gene expression in naïve *Nlrp3*$^{-/-}$ mice and most upregulated genes during *N. brasiliensis* infection. (Left) whole lung RNA was analysed by NanoString and volcano plots were generated to assess differential gene expression in naïve *Nlrp3*$^{-/-}$ mice compared to naïve WT mice. (Right) differential gene expression in WT mice infected with *N. brasiliensis* on day 7 post-infection compared to WT naïve mice. Data were from a single Nanostring run (n=2-4 per group). (**B**) IL-4 stimulation of BMDM cultures. BMDMs were generated from WT and *Nlrp3*$^{-/-}$ mice and stimulated for 24 hours with IL-4. Frequencies and gMFI are shown for Ym1 and RELM-α for CD11b$^+$ BMDMs analysed by flow cytometry. Data are pooled from 2 independent experiments (mean ± s.e.m.) from 2 individual experiments with 3-4 mice per group (per experiment).
